# Depression and its relationship with quality of life in frontline psychiatric clinicians during the COVID-19 pandemic in China: a national survey

**DOI:** 10.7150/ijbs.56037

**Published:** 2021-01-30

**Authors:** Hong-He Zhang, Yan-Jie Zhao, Chun Wang, Qinge Zhang, Hai-Yang Yu, Teris Cheung, Brian J. Hall, Feng-Rong An, Yu-Tao Xiang

**Affiliations:** 1 Department of Psychiatry, Xiamen Xianyue Hospital, Xiamen, China.; 2 Unit of Psychiatry, Institute of Translational Medicine, Faculty of Health Sciences, University of Macau, Macao SAR, China.; 3 Centre for Cognitive and Brain Sciences, & Institute of Advanced Studies in Humanities and Social Sciences, University of Macau, Macao SAR, China.; 4 The National Clinical Research Center for Mental Disorders & Beijing Key Laboratory of Mental Disorders Beijing Anding Hospital & the Advanced Innovation Center for Human Brain Protection, Capital Medical University, School of Mental Health, Beijing, China.; 5 Department of General Surgery, Beijing Liangxiang Hospital, Capital Medical University, Beijing, China.; 6 School of Nursing, Hong Kong Polytechnic University, Hong Kong SAR, China.; 7 Global and Community Mental Health Research Group, New York University (Shanghai), Shanghai PR China.; 8 School of Global Public Health, New York University, NY, USA.

**Keywords:** COVID-19, depression, psychiatric clinician, quality of life, prevalence

## Abstract

This was a national survey that determined the prevalence of depressive symptoms (depression thereafter) and its relationship with quality of life (QOL) in frontline clinicians working in psychiatric hospitals in China during the COVID-19 pandemic. Depression and QOL were assessed using the Patient Health Questionnaire nine items (PHQ-9) and the World Health Organization Quality of Life Questionnaire-brief version (WHOQOL-BREF), respectively. Multivariable logistic regression analyses and analysis of covariance were used. A total of 10,516 frontline clinicians participated in this study, of which, 28.52% (n=2,999) met screening criteria for depression. Compared to those without depression, clinicians with depression had a lower quality of life (*F*_(1, 10515)_ =2874.66, *P*<0.001). Higher educational level (*OR*=1.225, *P*=0.014), if the number of COVID-19 patients in the hospital catchment area surpassed 500 (*OR*=1.146, *P*=0.032), having family/friends/colleagues who were infected (*OR*=1.695, *P*<0.001), being a current smoker (*OR*=1.533, *P*<0.001), and longer working hours (*OR*=1.020, *P*=0.022) were independently associated with higher risk of depression. Living with family members (*OR*=0.786, *P*<0.001), and being junior clinicians (*OR*=0.851, *P*=0.011) were independently associated with lower odds of depression. The results showed that depression was common in frontline psychiatric clinicians during the COVID-19 pandemic. Timely assessment and effective interventions of depression for frontline clinicians in psychiatric hospitals were warranted.

## Introduction

The coronavirus disease 2019 (COVID-19) was first reported in China, and then was rapidly found in more than 200 countries and territories 1. To reduce rapid transmission, mass quarantine measures were adopted. Many public services including public transports were suspended 2. Such containment measures were associated with a range of psychological problems, such as anxiety, panic disorder, and depression 3-5. For instance, one study found that depressive symptoms (depression thereafter; 30.3%), anxiety (36.4%) and stress symptoms (32.1%) were common in the general population during the COVID-19 outbreak 6. With the rapid increase in the number of confirmed and suspected COVID-19 cases and insufficient preventive measures and personal protective equipment, uncertainty about the new virus and the known high risk of infection may have triggered common mental health problems in frontline health professionals 7. A survey reported that the prevalence of depressive symptoms was 50.4% and anxiety symptoms was 44.6% in health professionals who had previous exposure with COVID-19 8. Another study found that medical professionals reported insomnia (38.4%), anxiety (13.0%), depression (12.2%), and obsessive-compulsive symptoms (5.3%) during the COVID-19 outbreak 9.

Compared to those working in general hospitals, clinicians in psychiatric hospitals are presumed to work in a highly stressful clinical environment which makes them more vulnerable to higher risk of mental health problems 10, 11. Patients with severe mental illness in psychiatric hospitals often live in a crowded clinical environment compared to patients residing in general hospitals. Psychiatric patients may have limited intellectual ability / capacity to comply with infection control measures to protect themselves due to their poor mental health status and active symptomatology. Consequently, psychiatric patients are at heightened risk of susceptibility of the disease during the COVID-19 outbreak 12. Indeed, hundreds of psychiatric inpatients and many mental health professionals were infected with COVID-19 in China, and South Korea 13, 14. All of these aforementioned factors could increase the likelihood of mental health problems, particularly depression, among frontline clinicians in psychiatric hospitals since they have the most frequent direct contact with psychiatric patients on a daily basis. Aside from their heavy workload, clinicians may also experience fear of contagion and transmitting the virus to their family members and colleagues. Quality of life (QOL) is a widely used comprehensive health outcome measure involving many aspects such as physical and mental health, family relationship, education, employment, sense of security 15. Psychiatric problems particularly depression are negatively associated with QOL 16-18.

To alleviate the negative effect of depression on mental wellbeing and quality of care, it is essential to explore the pattern of depression in frontline psychiatric clinicians during the COVID-19 pandemic. However, to date, no relevant studies have been published, which gave us the impetus to conduct a large-scale study to examine the prevalence of depression and its relationship with QOL in frontline psychiatric clinicians in China.

## Methods

### Study design

This national, anonymous survey was carried out from 15 to 20 March, 2020. Considering the risk of infection, face-to-face assessments were not adopted. Instead, online assessment with the QuestionnaireStar program has been used based on convenience sampling as recommended previously in other epidemiological studies 19, 20. There were over 1 billion WeChat users in China and it has been widely used in continuing education for psychiatric clinicians. With the help of the Psychiatric Working Committee of the Chinese Nursing Association and the Chinese Society of Psychiatry, the Quick Response code (QR code) and the link to the assessment instruments were delivered to all member panels in each province by WeChat. The panel members then distributed the QR Code and the link to all psychiatric hospitals/units in their respective areas. Psychiatric clinicians in these hospitals/units completed the assessment on a voluntary basis. To be eligible, participants needed to be: 1) aged 18 years or older; 2) frontline clinicians (including psychiatric nurses, nursing assistants and psychiatrists) working in psychiatric departments/hospitals during the COVID-19 pandemic; The University of Macau Research Ethics Panel approved the study protocol, and all participants provided informed consent.

### Assessment tools

Demographic and clinical data were collected, including gender, age, marital status, educational level (e.g., primary and secondary school vs. college education and above). In addition, we also asked about the total number of local COVID-19 patients in participants' hospital catchment area (at the provincial level), whether they provided clinical services for COVID-19 patients, and having family, friends, or colleagues infected with the COVID-19.

We administered the Patient Health Questionnaire nine items (PHQ-9) to measure the presence of depression 21 , with each item scoring from “0” (not at all) to “3” (almost every day) 22. Having a PHQ-9 total score of ≥5 was considered 'having depression' 23. Specifically, a PHQ-9 total score of 5-9 indicated 'mild depression', 10-14 'moderate depression', 15-19 'moderately severe depression' 21. The total score ranged from 0-27. QOL was evaluated with the sum of the first two items (overall QOL) of the World Health Organization Quality of Life Questionnaire-brief version (WHOQOL-BREF) that has been validated in Chinese populations 24, 25. Higher total scores indicated higher QOL 26.

### Statistics

Data were analyzed with SPSS version 20.0. Comparisons between clinicians with and without depression in demographic and clinical factors were performed using Chi-square tests, independent samples t-tests, and Wilcoxon rank sum tests, as appropriate. Independent associated factors of depression were examined in multivariable logistic regression analyses using the “enter” method. Depression was the dependent variable, and all variables with a *P* value of < 0.05 in the univariate analysis were included as independent variables. The overall QOL between clinicians with and those without depression was compared with analysis of covariance. Significance level was set at 0.05 (two-tailed).

## Results

Altogether, 10,516 frontline clinicians fulfilled the entry criteria and participated in this study. The PHQ-9 mean score was 3.27 (*SD*=4.29). Of the frontline clinicians, 28.52% (95% *CI*: 27.66%-29.38%; 2,999/10,516) suffered from depression. Among the 2,999 clinicians with depression, 20.3% (2,137/10,516) reported mild depression, 5.5% (583/10,516) moderate depression, 1.8% (187/10,516) moderately severe depression, and 0.9% (92/10,516) severe depression. Demographic characteristics of the whole sample separated by depression were presented in Table 1.

**Figure 2:**
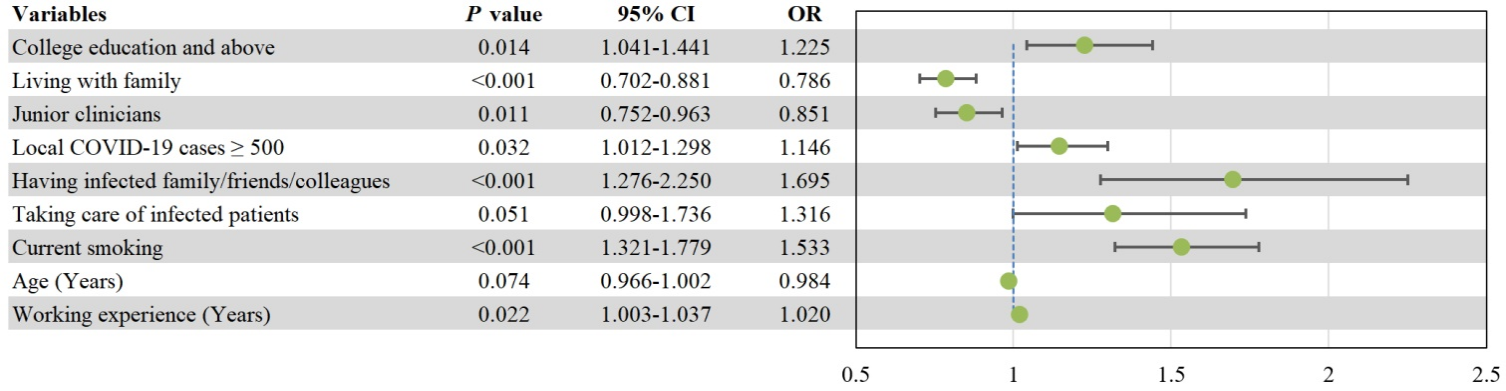
Independent correlates of depression by multiple logistic regression analysis. CI: confidential interval; OR: odds ratio; COVID-19: coronavirus disease 2019.

Table 1 shows that depression was significantly associated with age, educational level, living with family, clinicians' rank, and length of working experience, current smoking behavior, total number of local COVID-19 patients, having family/friends/colleagues who were infected, direct patient care of infected patients. ANCOVA revealed that clinicians with depression had lower QOL compared with those without depression (*F*_(1, 10515)_ =2874.66, *P*<0.001). Multivariable analysis found that higher educational level (*OR*=1.225, *P*=0.014), if the total number of local COVID-19 patients in residence area surpassed 500 (*OR*=1.146, *P*=0.032), having infected family/friends/colleagues (*OR*=1.695, *P*<0.001), current smoking behavior (*OR*=1.533, *P*<0.001), and longer working experiences (*OR*=1.020, *P*=0.022) were independently associated with higher odds of depression. Living with family (*OR*=0.786, *P*<0.001), and being junior clinicians (*OR*=0.851, *P*=0.011) were independently associated with lower risk of depression (Figure 2).

## Discussion

We found that 28.52% (95% *CI*: 27.66%-29.38%) of frontline clinicians reported depression, which was higher than the corresponding figures in most (e.g., 14.9% using the Depression Status Inventory (DSI) 27; 16.67% using the Self-rating Depression Scale (SDS) 28), but not all studies (e.g., 47.25% using the SDS 29) among psychiatric clinicians in China. Similar studies were also conducted in other countries, but only a few reported the prevalence of depression among clinicians in psychiatric hospitals. A study in Japan using the Center for Epidemiologic Studies for Depression Scale (CES-D) 30 showed that depression among male and female psychiatric clinicians were 36.4% and 37.2%, respectively. Nonetheless, direct comparisons should be conducted with caution due to different measurements and cut-off values used to assess depression between studies.

Frontline clinicians play a pivotal role in health services by rendering clinical care, advocating health education, and contributing to rehabilitation 31, 32. Compared with their counterparts working in other specialties, clinicians in psychiatric hospitals are more prone to suffer from psychological distress, such as burnout and depression because psychiatric patients may exhibit severe psychiatric symptoms, such as aggressive behaviors and suicidality 33. In addition, inadequate health resources are persistent problems in psychiatric hospitals in China 34, 35. More significantly, stigma still exists towards the field of psychiatry in China, and thus, clinicians working in psychiatric hospitals tend to have a relatively low social status 36.

Clinicians in psychiatric hospitals might end up with excessive workload during the COVID-19 pandemic as they were the first-line staff responsible for taking care of their inpatients 33. To make the situation worse, many COVID-19 patients suffered from psychiatric comorbidities 37, as such, psychiatric response teams were urgently established in many areas and provided services for these infectious patients with psychiatric symptoms in designated infectious hospital 38, 39, which may have further lowered the nurse-patient ratio in psychiatric hospitals. All these factors could immediately elevate the likelihood of depression among clinicians working in psychiatric hospitals.

In this study, clinicians with higher educational level and with more clinical experience in senior ranks reported a higher prevalence of depression. Apart from a heavy clinical workload, senior clinicians and experienced staff with higher educational backgrounds are primarily responsible for supervision of junior colleagues and conducting research 11. These senior clinicians are also more likely to provide clinical service to patients with severe psychiatric symptoms due to their clinical expertise 40. Their multi-functional role and extensive scope of service provision alongside with preceptorship and research responsibilities may trigger higher levels of stress among senior clinicians, resulting in a higher risk of depression.

As expected, clinicians who lived in greatly affected areas by COVID-19 (e.g., with ≥ 500 COVID-19 patients), had family, friends or colleagues who were infected, and direct care of infected patients had higher likelihood of depression due to fear of infection, stress, and anxiety. All clinicians underwent a minimum of two-week's quarantine following their care for infected patients, which could further increase psychological distress due to fear of contagion and infect others. Clinicians living with families usually had better social support, and this could effectively lower the likelihood of depression and improve wellbeing 41. We also found that smoking behavior was related to higher risk of depression, confirming previous findings 42, 43.

Clinicians with depression reported lower QOL, which supports previous findings in other populations 44, 45. According to the distress/protection QOL model 46, QOL was determined by distressing (e.g., physical and mental distress) and protective factors (e.g., better health status). Due to the somatic and psychiatric symptoms associated with depression and their impact on daily life and functional outcomes, it was unsurprising that depressed clinicians had lower QOL.

Several limitations should be noted. First, causality between demographic and clinical variables and depression cannot be examined because of the cross-sectional design. Second, data were collected via an online self-reported survey; therefore, participants may have misunderstood some survey questions. Third, some factors related to depression in frontline clinicians, such as history of psychiatric illness, social support, negative life events, and type of clinical training, were not examined. Fourth, the classification of frontline clinicians working in psychiatric hospitals such as psychiatric nurses, nursing assistants and psychiatrists was not made as this could not be verified in an online survey. Therefore, direct comparisons of depression between different subpopulations could not be performed.

In conclusion, depression was prevalent in Chinese psychiatric clinicians during the COVID-19 pandemic. Routine assessment and effective treatment of depression for frontline clinicians is warranted. To reduce the risk of mental health problems among psychiatric clinicians in future public health crises, health authorities could develop guidelines and expert consensus to address occupational health and safety conditions, and crisis psychological intervention and counseling for frontline health professionals 47. In addition, it would be helpful to assist their families to handle daily life requirements and securing their financial status. The establishment of timely psychological services and the provision of on-site mental health services for frontline health professionals should also be considered.

## Figures and Tables

**Figure 1:**
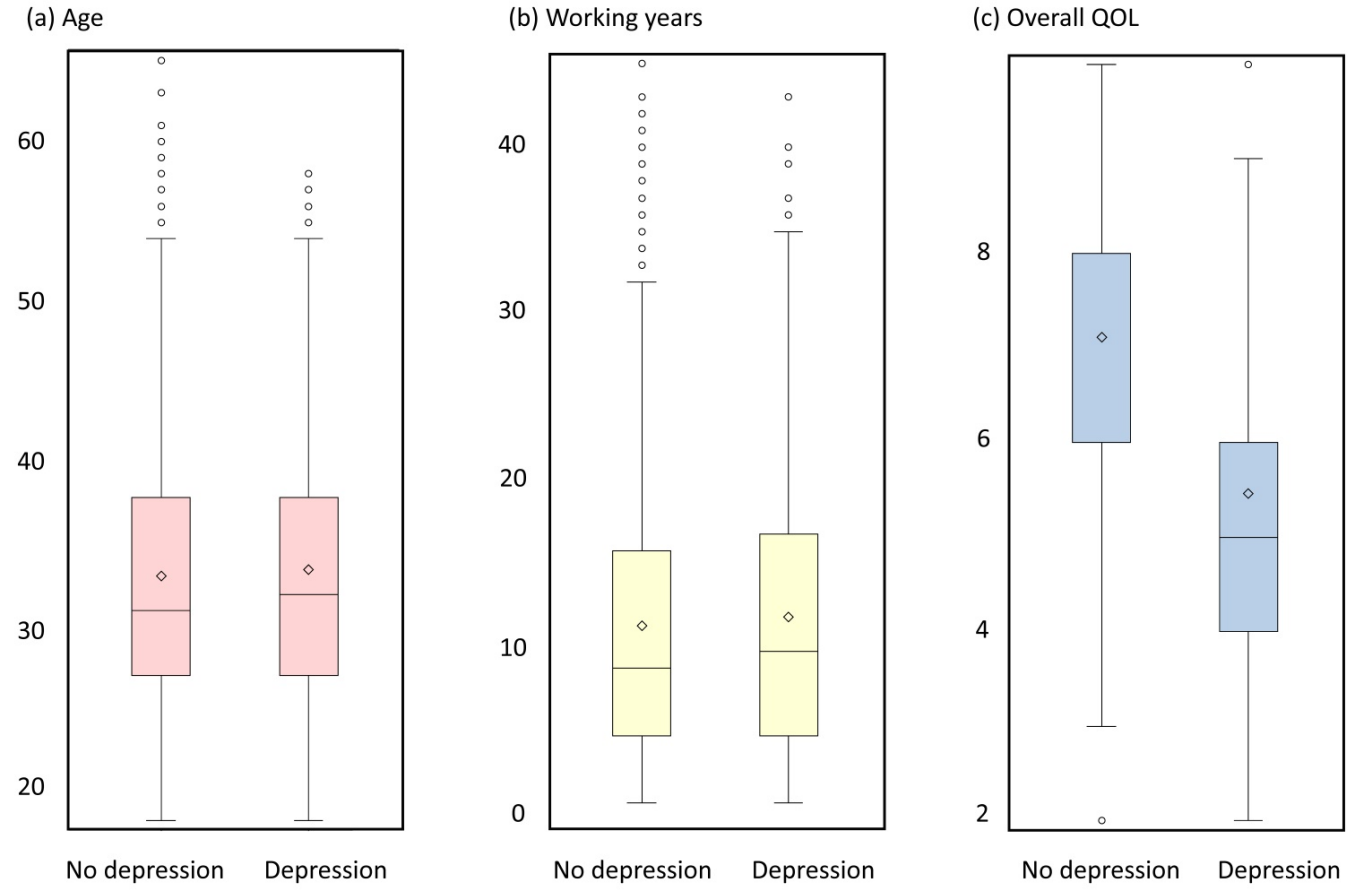
Box plots of age, working years and overall QOL.

**Table 1:** Demographic characteristics of participants

Variables	Total (N=10,516)		No depression (N=7,517)		Depression (N=2,999)		χ^2^	*df*	*P*
	N	%	N	%	N	%			
Male gender (ref = female)	1,635	15.5	1,145	15.2	490	16.3	1.999	1	0.157
Married (ref = not married)	7,273	69.2	5,231	69.6	2,042	68.1	2.260	1	0.133
College education and above	9,635	91.6	6,861	91.3	2,774	92.5	4.187	1	**0.041**
Living with family	8,629	82.1	6,234	82.9	2,395	79.9	13.740	1	**<0.001**
Junior clinicians (ref = senior)	7,341	69.8	5,326	70.9	2,015	67.2	13.652	1	**<0.001**
Experience of fighting SARS	948	9.0	666	8.9	282	9.4	0.771	1	0.380
Working in tertiary hospitals	6,564	62.4	4,713	62.7	1,851	61.7	0.873	1	0.350
Working in inpatient department	9,642	91.7	6,881	91.5	2,761	92.1	0.775	1	0.379
Shift duty clinicians	7,719	73.4	5,482	72.9	2,237	74.6	3.039	1	0.081
Local COVID-19 cases ≥ 500	1,361	12.9	927	12.3	434	14.5	8.709	1	**0.003**
Family/friends/colleagues infected	213	2.0	121	1.6	92	3.1	22.964	1	**<0.001**
Taking care of infected patients	235	2.2	146	1.9	89	3.0	10.317	1	**0.001**
Current smoking	854	8.1	542	7.2	312	10.4	29.294	1	**<0.001**
	*Mean*	*SD*	*Mean*	*SD*	*Mean*	*SD*	*t/Z*	*df*	*P*
Age (Years)	33.25	8.40	33.14	8.50	33.50	8.16	-1.974	10514	**0.048**
Working experience (Years)	11.66	9.10	11.51	9.21	12.04	8.83	4.71*	—	**<0.001**
Total QOL score	6.64	1.60	7.12	1.42	5.46	1.39	54.288	10514	**<0.001**

Bolded values: <0.05; SD: standard deviation; COVID-19: Corona Virus Disease 2019; SARS: Severe Acute Respiratory Syndrome; QOL: Quality of Life; * Wilcoxon rank sum test.

## Abbreviations

QOL: quality of life
PHQ-9: patient health questionnaire nine items
WHOQOL-BREF: world health organization quality of life questionnaire-brief version
COVID-19: coronavirus disease 2019
DSI: depression status inventory
SDS: self-rating depression scale
CES-D: center for epidemiologic studies for depression scale
